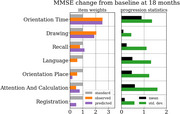# Understanding and Optimizing Composite Scores for Targeted Cohorts with Machine Learning

**DOI:** 10.1002/alz70859_104167

**Published:** 2025-12-25

**Authors:** Coco Kusiak, Jonathan R. Walsh, Run Zhuang

**Affiliations:** ^1^ Unlearn.AI, San Francisco, CA USA

## Abstract

**Background:**

Alzheimer’s disease (AD) affects multiple cognitive and functional domains, and clinical trials use a variety of assessments to measure impairment and the efficacy of new treatments. Because different items in assessments can vary substantially in terms of their sensitivity to changes in impairment at particular disease stages, composite scores are often noisy and difficult to power. Optimizing the selection and weighting of items in and across assessments is a strategy that can lead to greater statistical power and less patient burden, especially in Phase 2 trials where sponsors have greater flexibility in endpoint selection.

**Method:**

We leveraged a novel approach for optimizing composite scores, borrowing a method from portfolio optimization in finance called the efficient frontier. For a given cohort, the method identifies optimal linear combinations of items to maximize the expected control arm progression for a given target variance, and can be used to yield a globally optimal composite score. This can lead to substantial increases in statistical power. We applied this approach to five commonly used assessments in early AD: Alzheimer’s Disease Assessment Scale Cognitive subscale (ADAS‐Cog13), Mini Mental State Exam (MMSE), Clinical Dementia Rating (CDR), Functional Activities Questionnaire (FAQ), and the Alzheimer’s Disease Cooperative Study Activities of Daily Living (ADCS‐ADL). Together with digital twin predictions of item‐level progression, the efficient frontier can be used to understand and prospectively optimize composite scores for targeted cohorts.

**Result:**

Optimizing the composite score for an early AD population of participants demonstrated significant improvements in statistical power for certain endpoints, such as an 18% gain in power (from 50% to 68%) for ADAS‐Cog13 at 18 months. Combining results across assessments using digital twin predictions, we identified new composite scores that highlight a variety of cognitive domains, functional impairment, or a combination.

**Conclusion:**

This approach brings a novel tool to the task of endpoint selection, especially in early phase studies where signal detection is paramount. The method produces interpretable findings and can be applied on individual assessments or across assessments. Optimizing composite scores for specific cohorts allows sponsors to define endpoints more sensitive to treatment effects, reduce patient burden, and improve decision making in trials.